# Supplementary material for: Pressurized Hot Liquid Extraction with 15% v/v Glycerol-Water as An Effective Environment-Friendly Process to Obtain Durvillaea incurvata and Lessonia spicata Phlorotannin Extracts with Antioxidant and Antihyperglycemic Potential
Source: Antioxidants (Basel). 2021 Jul 10;10(7):1105. doi: 10.3390/antiox10071105 (PMC8301173; doi:10.3390/antiox10071105)
Supplement: Supplementary file 1 [file antioxidants-10-01105-s001.zip › antioxidants-1273359-supplementary/antioxidants-1273359-supplementary.pdf]

Supplementary Materials: The following are available online at www.mdpi.com/xxx/s1, Table S1: Three-way ANOVA for the effects of the species, alga part used, and collection zone on the PHLE extracts' TPC values, expressed as dry seaweed; Table S2: Three-way ANOVA for the effects of the species, alga part used, and collection zone on the PHLE extracts' DPPH values, expressed as dry seaweed; Table S3: Three-way ANOVA for the effects of the species, alga part used, and collection zone on the PHLE extracts' ORAC values, expressed as dry seaweed; Table S4: One-way ANOVA for the effect of the seaweed PHLE extract type on their IC_{50} values against α-glucosidase activity; Table S5: Multiple comparisons Tukey post hoc tests performed on the IC_{50} values of the PHLE extracts and acarbose against α-glucosidase activity; Table S6: Three-way ANOVA for the effects of the species, alga part used, and collection zone on the PHLE extracts' mannitol content, expressed as % dry weight seaweed; Table S7: Multiple comparisons Tukey post hoc tests performed on the PHLE extracts mannitol content means (expressed as % dry weight seaweed), per each studied factor: species, zone, and alga part used; Table S8: Three-way ANOVA for the effects of the species, alga part used, and collection zone on the As content of the seaweed samples, expressed as dry weight, Table S9: Multiple comparisons Tukey post hoc tests performed on the seaweed samples' As content, per each studied factor: species, zone, and alga part used.
