# Supplementary material for: Pressurized Hot Liquid Extraction with 15% v/v Glycerol-Water as An Effective Environment-Friendly Process to Obtain Durvillaea incurvata and Lessonia spicata Phlorotannin Extracts with Antioxidant and Antihyperglycemic Potential
Source: Antioxidants (Basel). 2021 Jul 10;10(7):1105. doi: 10.3390/antiox10071105 (PMC8301173; doi:10.3390/antiox10071105)
Supplement: Supplementary file 1 [file antioxidants-10-01105-s001.zip › antioxidants-1273359-supplementary/Supp. Table 6.pptx]

## Slide 1
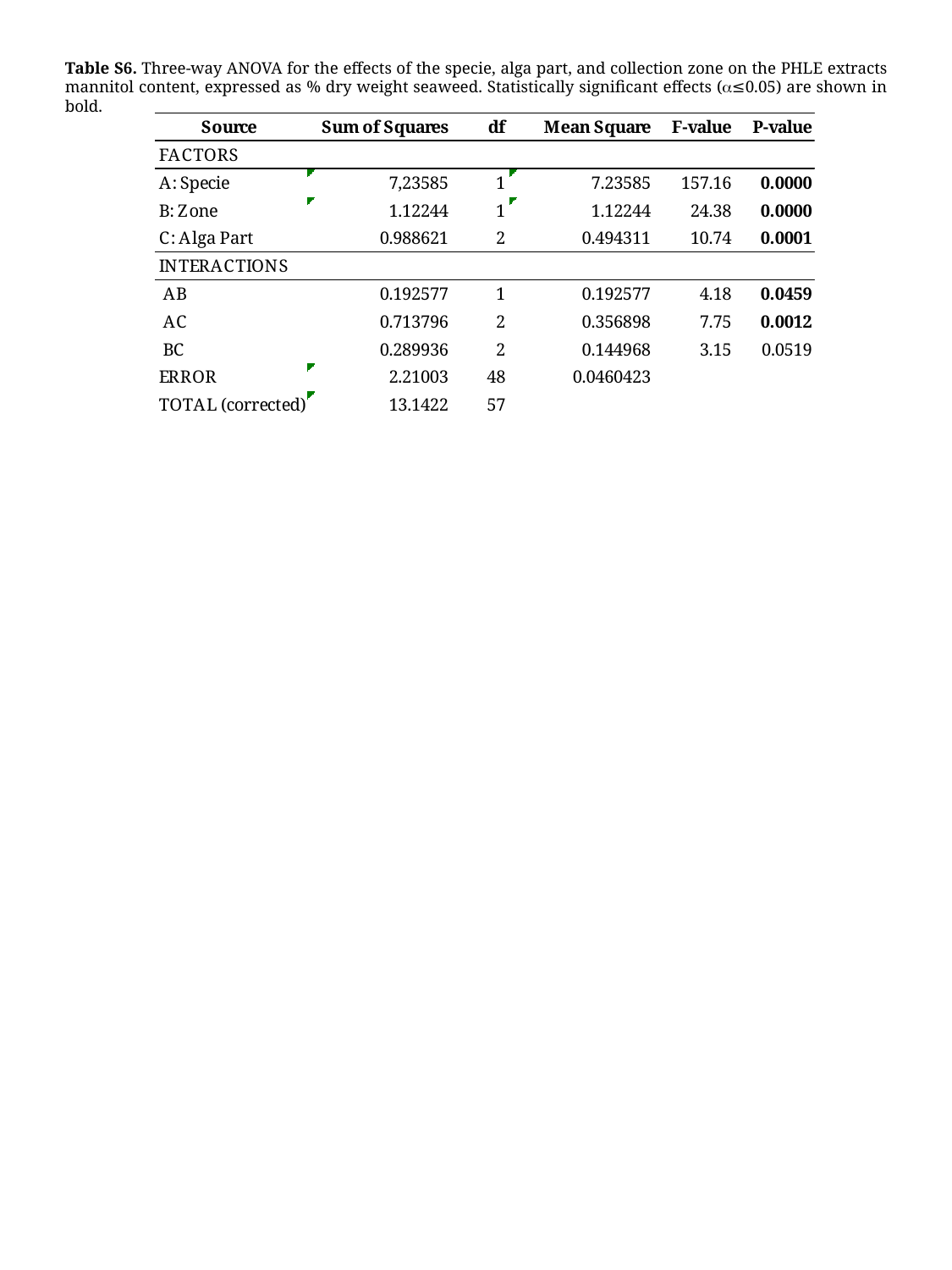

Table S6. Three-way ANOVA for the effects of the specie, alga part, and collection zone on the PHLE extracts mannitol content, expressed as % dry weight seaweed. Statistically significant effects (a≤0.05) are shown in bold.
